# The Mitophagy Vortex: Bridging Inflammatory, Oxidative, Hypoxic, and Aging Insults to Drive COPD Skeletal Muscle Dysfunction

**DOI:** 10.3390/biomedicines14102240

**Published:** 2026-10-03

**Authors:** Jian Li, Peijun Li, Xianli Zhao, Lele Jiang, Hui Li, Fuying Chen, Cai-tao Chen, Weibing Wu, Xiaodan Liu

**Affiliations:** 1 School of Rehabilitation Science, Shanghai University of Traditional Chinese Medicine, Shanghai 201203, China; lijianfitness@126.com (J.L.); lpj0227@163.com (P.L.); zhaoxianli16@126.com (X.Z.); 18803656753@163.com (L.J.); 15601734645@163.com (H.L.); 2 School of Exercise and Health, Shanghai University of Sport, Shanghai 200438, China; 18817512196@163.com; 3 Department of Rehabilitation Medicine, Shanghai Fourth People’s Hospital, School of Medicine, Tongji University, Shanghai 200434, China

**Keywords:** chronic obstructive pulmonary disease, skeletal muscle dysfunction, mitophagy, systemic inflammation, oxidative stress, hypoxia, aging

## Abstract

Skeletal muscle dysfunction is a frequent extrapulmonary complication of chronic obstructive pulmonary disease (COPD) that limits physical capacity and increases mortality. Systemic inflammation, oxidative stress, hypoxia, and aging are distinct in origin and signaling, yet each has been implicated in COPD muscle deterioration. Whether these four drivers converge on a common pathway remains unclear. This review proposes that mitophagy, the selective elimination of damaged mitochondria, serves as this hub. Rather than acting in isolation, the four drivers converge through two functionally distinct amplification axes. The inflammatory–oxidative axis escalates mitochondrial injury, while the hypoxic–aging axis progressively erodes lysosomal disposal capacity, lowering the threshold for quality-control failure. Once injury load outruns clearance capacity, retained damaged mitochondria release mtDNA and overproduce ROS, which amplify upstream inflammatory signals and further impair lysosomal function, a self-reinforcing disequilibrium in which each iteration amplifies the next. This architecture, which we term the mitophagy vortex, implies that the four factors do not add linearly but multiply through a common quality-control node, explaining why single-target interventions have consistently failed. The framework predicts that restoring lysosomal acidification or autophagic flux, rather than neutralizing individual cytokines or ROS, may carry greater therapeutic weight once the vortex is established. Targeting the mitophagy machinery at multiple levels, including its receptors, enzymes, and lysosomal function, represents a mechanistically coherent strategy for breaking the pathological cycle.

## 1. Introduction

Chronic obstructive pulmonary disease (COPD) remains the third leading cause of death globally, yet its morbidity extends far beyond the airways [1,2]. Among the numerous extrapulmonary complications, skeletal muscle dysfunction stands out as a robust and independent predictor of poor prognosis [3,4]. This complication, characterized by reduced strength, early fatigue, and muscle atrophy, is consistently linked to increased hospitalization and all-cause mortality in COPD patients [5,6]. Current pharmacotherapies, including bronchodilators and anti-inflammatory regimens, effectively control pulmonary obstruction but consistently fail to halt or even reverse the progressive decline [7]. This therapeutic void implies that the mechanisms driving COPD-induced skeletal muscle wasting are largely distinct from, and poorly mirrored by, the airway-centric inflammatory model.

However, attributing skeletal muscle dysfunction solely to the spillover of pulmonary pathology is insufficient to explain its full complexity. Current evidence supports the notion that COPD skeletal muscle dysfunction is now recognized as a multifactorial process [8,9,10,11]. Systemic inflammation, oxidative stress, hypoxia, and accelerated aging are recognized as the core drivers of this pathological state. These factors are thought to act in concert through multiple downstream mechanisms, including activation of the ubiquitin–proteasome system, enhancement of autophagy–lysosomal pathways, suppression of protein synthesis, and interference with satellite cell-mediated muscle regeneration, collectively promoting myofiber atrophy and functional decline [12].

Nevertheless, whether these four factors act independently on skeletal muscle or converge upon a shared molecular hub that amplifies their individual effects remains largely unknown. The conventional research paradigm has favored a reductionist approach, interrogating each pathogenic factor as an isolated therapeutic target. The translational outcomes of this strategy, however, have been largely disappointing [13,14,15]. Clinical trials attempting to neutralize single inflammatory cytokines, such as TNF-α, have failed to reverse muscle wasting in COPD patients [13,14]. Likewise, supplementation with broad-spectrum antioxidants has not yielded consistent or clinically meaningful improvements in muscle endurance or exercise capacity [15]. These recurring negative findings argue against a simple, linear model of causation. Instead, they suggest that the four factors are engaged in a complex crosstalk, mutually reinforcing one another through positive feedback loops [16]. Under this framework, intervening in any single pathway would be inherently insufficient to reset the pathological network. Therefore, identifying and validating a central molecular hub that integrates the four pathogenic drivers has become a critical priority for understanding the pathogenesis of COPD-induced skeletal muscle dysfunction and for developing novel, mechanism-based therapeutic strategies.

Among the numerous mechanisms proposed to explain the convergence of these pathogenic factors, mitophagy has attracted growing interest as a potential mechanism linking COPD to skeletal muscle pathology [17]. Mitophagy is defined as the selective autophagic elimination of damaged or dysfunctional mitochondria. Under physiological conditions, mitophagy serves as a critical quality control mechanism that maintains mitochondrial network integrity, regulates cellular bioenergetics, and prevents the accumulation of pro-inflammatory mitochondrial constituents [18,19]. However, the prevailing literature has largely cast mitophagy as a passive victim of pathogenic stimuli, a downstream executor that merely clears the debris generated by oxidative injury or inflammatory stress. This view is incomplete. Recent evidence suggests that mitophagy dysfunction is not merely a consequence but can actively propagate and amplify pathological signals, feeding back to exacerbate inflammation, enhance oxidative bursts, impair hypoxic adaptation, and accelerate aging phenotypes [19,20].

Here, we synthesize the emerging evidence on mitophagy in COPD skeletal muscle and propose a working model, the mitophagy vortex, in which mitophagy failure entrains the four pathogenic drivers into a self-sustaining, spiraling network. Our analysis begins with a condensed overview of the mitophagy machinery, with particular attention to features relevant to skeletal muscle physiology. We then examine two interconnected amplification mechanisms through which mitophagy dysfunction reinforces the pathological network. In the first, defective mitophagy fuels a reciprocal inflammatory–oxidative tandem; in the second, hypoxic and aging contexts undermine the autophagic clearance capacity, thereby lowering the threshold for mitochondrial quality control failure. Finally, we consider the therapeutic implications of this integrative framework and identify the critical knowledge gaps that must be addressed before mitophagy-directed interventions can be translated into clinical benefit for COPD patients.

## 2. Mitophagy Mechanisms

**Figure 1:**
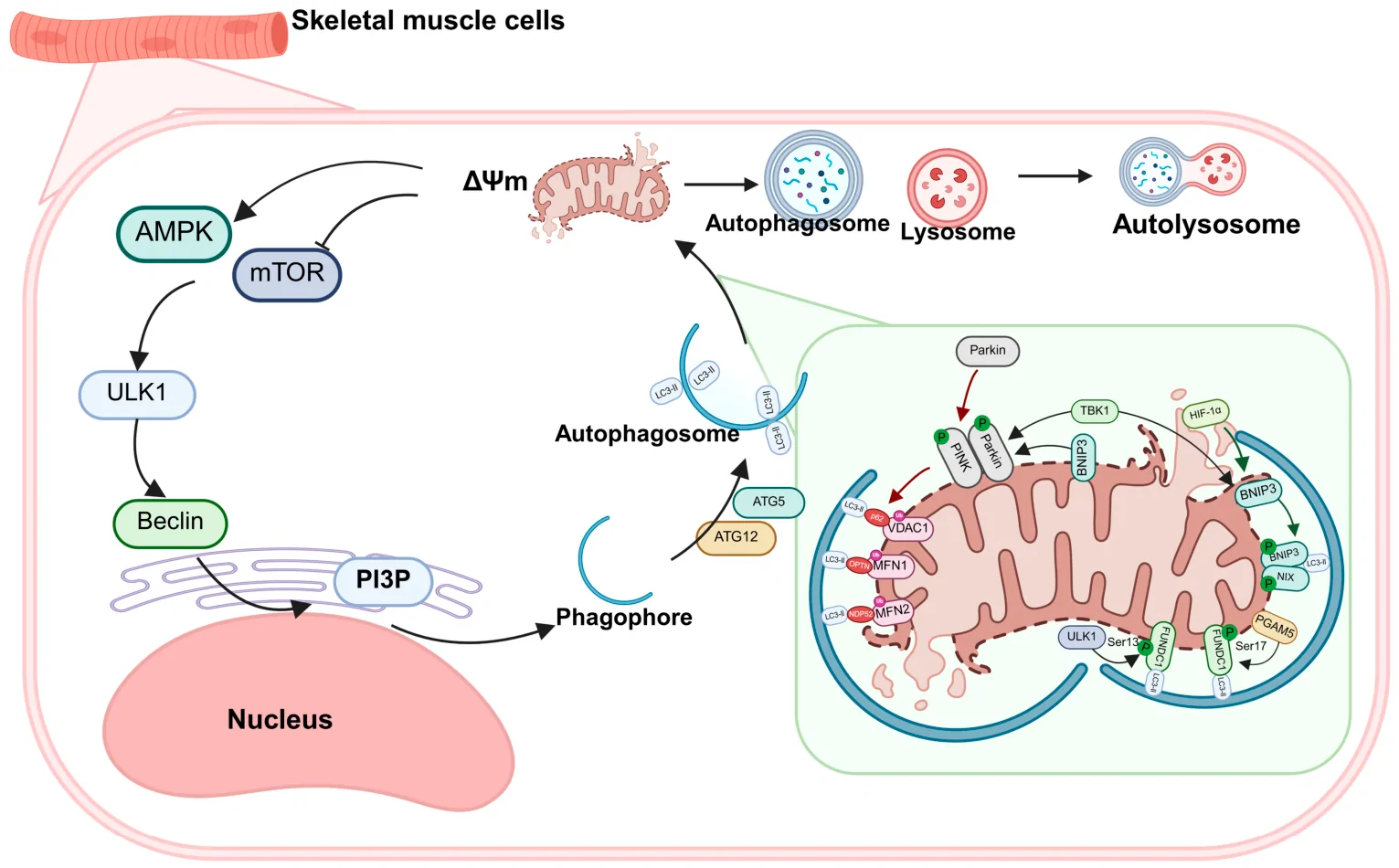
Mitophagy mechanism. Mitophagy is triggered by mitochondrial depolarization (ΔΨm loss), which activates AMPK and inhibits mTORC1, leading to ULK1 complex activation. ULK1 releases Beclin1 to promote PI3P production and phagophore formation. The phagophore expands and closes through ubiquitin-like conjugation cascades, resulting in the formation of an autophagosome. Damaged mitochondria are recognized via ubiquitin-dependent (PINK1/Parkin) and ubiquitin-independent (BNIP3/NIX, FUNDC1) pathways. The autophagosome fuses with a lysosome to form an autolysosome, where mitochondria are degraded. This figure panel was created in BioRender. Li, J. (2026) https://BioRender.com/l489zgr (accessed on 28 September 2026). Abbreviations: ΔΨm, mitochondrial membrane potential; AMPK, AMP-activated protein kinase; mTOR, mechanistic target of rapamycin; ULK1, Unc-51-like kinase 1; Beclin, coiled-coil myosin-like BCL2-interacting protein; PI3P, phosphatidylinositol 3-phosphate; ATG, autophagy-related gene; LC3-II, lipidated form of microtubule-associated protein 1 light chain 3; PINK, PTEN-induced putative kinase; Parkin, E3 ubiquitin-protein ligase; VDAC, voltage-dependent anion channel; MFN, mitofusin; BNIP3, BCL2-interacting protein 3; NIX, BNIP3-like protein (BNIP3L); FUNDC1, FUN14 domain-containing protein 1; HIF-1α, hypoxia-inducible factor 1-alpha; PGAM5: phosphoglycerate mutase family member 5; ULK1: Unc-51-like kinase 1; TBK1: TANK-binding kinase 1; P, phosphorylation; Ser, serine; Ub, ubiquitin; p62, sequestosome-1 (SQSTM1); OPTN, optineurin; NDP52, nuclear dot protein 52 kDa.

Mitophagy, the selective autophagic disposal of damaged mitochondria, is the primary mechanism by which cells safeguard mitochondrial network integrity and prevent the accumulation of dysfunctional organelles (Figure 1). In skeletal muscle, where mitochondrial turnover is particularly active due to high and fluctuating energy demands, mitophagy plays a non-redundant role in maintaining contractile function and preventing the accumulation of dysfunctional organelles [21,22]. The core autophagosome formation machinery, including the ULK1 complex, the Beclin1-VPS34 nucleation module, and the ATG12-ATG5-ATG16L1/LC3 lipidation cascades, has been extensively characterized elsewhere [23,24,25] and is not reiterated here. Instead, this section focuses on the molecular recognition mechanisms that tag-damaged mitochondria for clearance, with an emphasis on the pathways most extensively studied in the context of COPD skeletal muscle, namely the PINK1/Parkin axis and the receptor-mediated pathways involving BNIP3/NIX and FUNDC1. The subsequent autophagosome–lysosome fusion step is briefly addressed to highlight its relevance to the autophagic flux failure that emerges as a key theme in later sections.

Two principal pathways mediate the recognition of damaged mitochondria by the autophagosomal membrane: the ubiquitin-dependent PINK1/Parkin axis and the ubiquitin-independent receptor-mediated pathways involving BNIP3/NIX and FUNDC1. Under physiological conditions, PINK1 is rapidly degraded; upon mitochondrial depolarization, PINK1 accumulates on the outer mitochondrial membrane, phosphorylates Parkin at Ser65, and unleashes its E3 ubiquitin ligase activity [26,27]. Activated Parkin ubiquitinates multiple outer membrane proteins, including VDAC1 and mitofusins (MFNs), and the resulting ubiquitin chains recruit autophagy adaptors such as p62, OPTN, and NDP52, which simultaneously bind LC3 on the phagophore to tether damaged mitochondria for clearance [28,29]. In parallel, receptor-mediated mitophagy operates through outer membrane proteins that directly engage LC3 via their LC3-interacting region (LIR) motifs. BNIP3 and NIX are transcriptionally upregulated by HIF-1α under hypoxic conditions and, upon phosphorylation by upstream kinases, bind LC3 with high affinity to mediate ubiquitin-independent mitochondrial clearance [30,31]. FUNDC1, another outer membrane receptor, is regulated by a phosphorylation switch: under normoxia, its LIR motif is masked by inhibitory phosphorylation; upon hypoxic stress, dephosphorylation at Ser13 by PGAM5 and phosphorylation at Ser17 by ULK1 expose the LIR motif and trigger rapid mitophagy [32,33]. These two pathways are not strictly separate but exhibit cross-regulation, exemplified by TBK1-mediated phosphorylation of both Parkin and OPTN, as well as BNIP3-mediated stabilization of PINK1 [34,35].

Recognition and engulfment of damaged mitochondria, however, do not guarantee successful mitophagy. The final and equally critical step is the fusion of the autophagosome with a lysosome, a process orchestrated by the Rab7 GTPase, the homotypic fusion and protein sorting (HOPS) tethering complex, and the soluble NSF attachment protein receptor (SNARE) machinery, ultimately generating an autolysosome wherein the encapsulated mitochondrion is degraded and its constituents recycled [36]. This fusion step determines whether mitophagy is productively executed or remains abortive, a distinction captured by the concept of autophagic flux, the continuous throughput from cargo sequestration to lysosomal degradation. Crucially, autophagic flux cannot be inferred solely from static measurements of LC3-II or p62 levels, a caveat particularly relevant to human muscle biopsy studies [37]. Under pathological conditions such as those encountered in COPD, the lysosomal compartment is subjected to substantial stress, as elaborated in subsequent sections. Impairments in lysosomal acidification, autophagosome–lysosome fusion, or lysosomal enzyme activity can transform an otherwise compensatory mitophagic response into a pathological bottleneck, where mitochondria are sequestered but not cleared. Thus, the distinction between mitophagy initiation and flux completion emerges as a critical determinant of whether the process confers protection or contributes to pathology in skeletal muscle.

## 3. Multifactorial Mechanisms of Skeletal Muscle Dysfunction in COPD

Not all COPD patients develop skeletal muscle dysfunction; approximately 40% show significant manifestations [38]. This clinical heterogeneity, compounded by the lack of uniform assessment criteria [39,40], has led to considerable variability in reported incidence across studies. More importantly, it highlights a fundamental biological question that remains inadequately addressed, namely what determines this differential susceptibility. The multifactorial nature of this dysfunction is well established, involving systemic inflammation, oxidative stress, hypoxia, and aging [10,41]. Despite extensive documentation of these individual factors, how they interact with one another and whether they converge on a common molecular node to drive muscle pathology remain largely unclear. Here, we re-examine these four established pathogenic drivers through the lens of mitophagy, asking not only how they damage muscle fibers but also how they collectively converge on and disrupt the mitochondrial quality control machinery. By mapping this convergence, we aim to identify the central regulatory node that serves as a critical therapeutic target for COPD skeletal muscle dysfunction.

### 3.1. Systemic Inflammation

**Figure 2:**
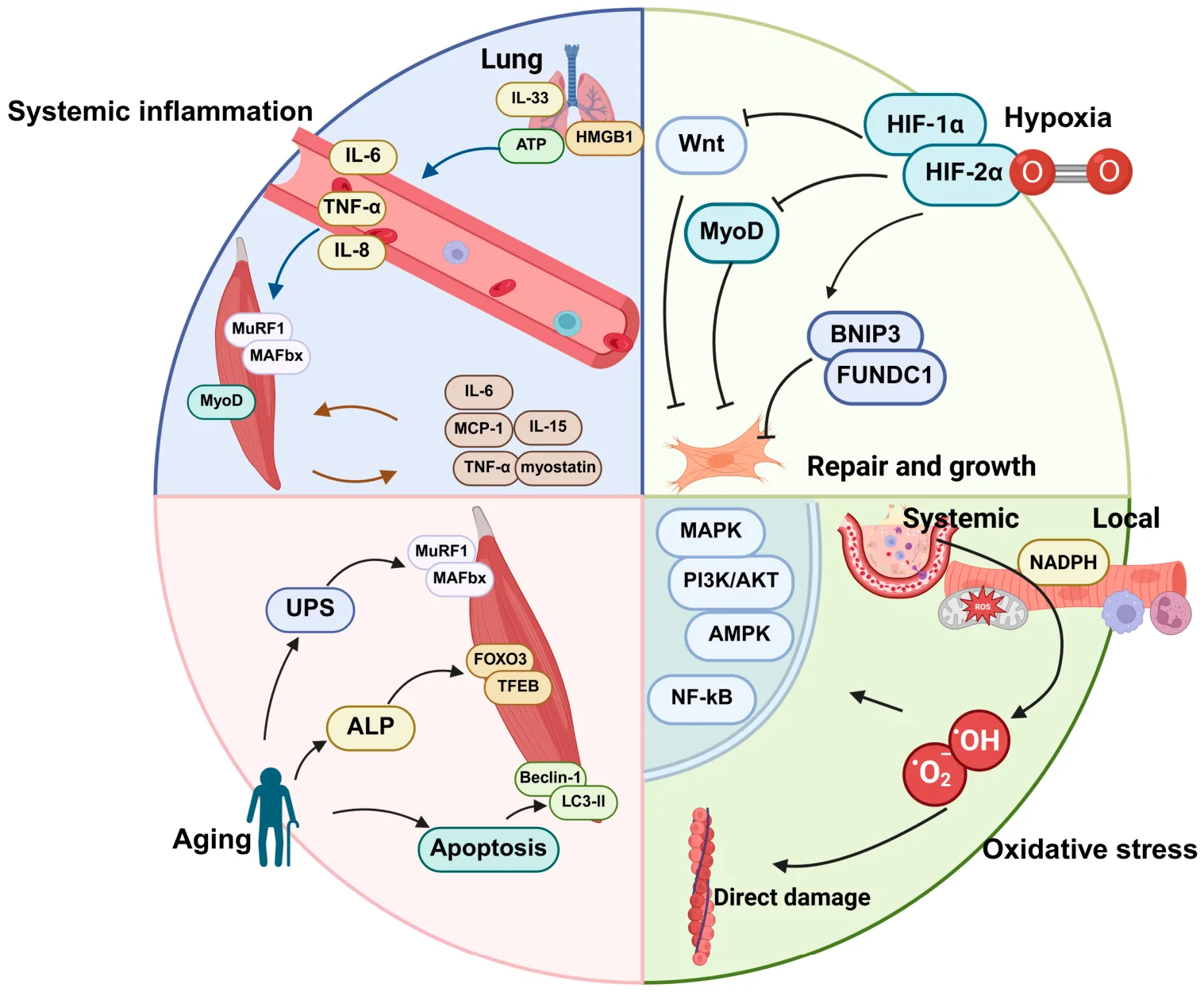
Multifactorial pathogenesis of skeletal muscle dysfunction in COPD. Skeletal muscle dysfunction in COPD results from the convergence of four pathogenic drivers: systemic inflammation, oxidative stress, hypoxia, and aging. Systemic inflammation damages skeletal muscle directly via inflammatory mediators and indirectly by promoting myokine secretion that amplifies the inflammatory cascade. Oxidative stress induces contractile protein oxidation, excitation–contraction uncoupling, and activation of proteolytic systems. Hypoxia suppresses protein synthesis, promotes autophagy, and impairs satellite cell proliferation. Aging activates the ubiquitin–proteasome and autophagy–lysosome pathways, accelerating protein degradation and mitochondrial dysfunction. Collectively, these four drivers converge to promote myofiber atrophy, impaired regeneration, and functional decline. This figure panel was created in BioRender. Li, J. (2026) https://BioRender.com/l489zgr (accessed on 28 September 2026). Abbreviations: ROS, reactive oxygen species; IL, interleukin; TNF, tumor necrosis factor; HMGB1, high mobility group box 1; ATP, adenosine triphosphate; MCP, monocyte chemoattractant protein; MuRF1, muscle RING finger 1; MAFbx, muscle atrophy F-box; MyoD, myogenic differentiation; HIF, hypoxia-inducible factor; BNIP, BCL2/adenovirus E1B 19 kDa-interacting protein; FUNDC1, FUN14 domain-containing protein 1; MAPK, mitogen-activated protein kinase; Wnt, wingless-related integration site; PI3K/AKT, phosphatidylinositol 3-kinase/protein kinase B; UPS, ubiquitin–proteasome system; ALP, autophagy–lysosome pathway; FOXO3, forkhead box O3; TFEB, transcription factor EB; Beclin, coiled-coil myosin-like BCL2-interacting protein; LC3-II, microtubule-associated protein 1 light chain 3-II; NF-κB, nuclear factor kappa-light-chain-enhancer of activated B cells.

Systemic inflammation in COPD involves a complex, multi-level interplay among immune cells, myofibers, and distal organs. Unlike local pulmonary inflammation, which cannot readily account for the remote effects on peripheral muscles, systemic inflammation has been established as a key driver of skeletal muscle dysfunction in COPD [42,43,44]. In this context, the contribution of inflammation is not limited to circulating cytokines acting directly on myofibers; it also involves the active participation of muscle tissue itself in amplifying inflammatory signals, as well as the engagement of immune cells whose functions are modulated by mitochondrial quality control. This multi-cellular perspective forms the basis for understanding how inflammation intersects with mitophagy in the pathogenesis of COPD skeletal muscle dysfunction (Figure 2).

#### 3.1.1. Pulmonary Inflammation Induces Systemic Inflammation

Airway inflammation in COPD is characterized by increased infiltration of macrophages, neutrophils, and lymphocytes, which secrete a network of pro-inflammatory cytokines [45]. The prevailing view holds that these local inflammatory mediators can translocate into the circulation through a mechanism known as the “spillover” hypothesis [46,47]. Chronic smoke exposure increases airway permeability [48], and combined with structural alterations such as goblet cell hyperplasia and cilia loss, facilitates the translocation of pathogens and damage-associated molecular patterns (DAMPs) across the epithelial barrier [49]. These stimuli, including IL-33, HMGB1, and ATP, activate dendritic cells and macrophages, triggering NF-κB/NLRP3 signaling and the release of IL-6, IL-8, and TNF-α into the bloodstream [50,51]. Subsequent studies have progressively established a stable link between inhalation-induced pulmonary inflammation and systemic inflammation [52,53]. Alveolar macrophages serve as central sensors in this process, linking local inflammatory activation to systemic inflammatory responses [54,55,56,57]. Clinical evidence further reinforces this pulmonary-to-systemic spread, with elevated circulating IL-1 and IL-6 observed during acute respiratory infections [58,59], and persistent low-grade inflammation during remission [60,61,62].

#### 3.1.2. The Transformation Role of Skeletal Muscles in Systemic Inflammation

Myokines generated during skeletal muscle contraction constitute another important source of systemic inflammation in COPD, as detailed in a previous review [8]. Cytokines secreted by skeletal muscle act in autocrine, endocrine, or paracrine manners on tissues such as bone, brain, liver, and muscle, thereby contributing to systemic inflammation [63]. This places skeletal muscle not merely as a passive victim of systemic inflammation but as an active participant. The involvement of skeletal muscle in systemic inflammation can be understood as a sequential process. Upon sensing pro-inflammatory signals that spill over into the circulation, including IL-6, TNF-α, and ROS [64,65], these factors bind to specific receptors on the myocyte membrane, such as gp130, TNFR1, and TLR4, converting extracellular inflammatory stimuli into intracellular signals. In response, overexpression of these membrane receptors further activates JAK/STAT3 and NF-κB signaling pathways, driving myofibers to secrete large amounts of myokines including IL-6, TNF-α, MCP-1, IL-15, myostatin, VEGF-A, and irisin [66,67], thereby amplifying the inflammatory cascade. This stage marks a functional transition of skeletal muscle from a contractile organ to a secretory organ. Once released into the circulation, these myokines act on remote target organs. For example, myostatin reaches the liver and activates SMAD2/3 signaling, inducing the synthesis of CRP, fibrinogen, and serum amyloid A, thereby perpetuating systemic inflammation [68]. Similarly, exogenous IL-6 activates STAT3 signaling and promotes inflammatory processes; elevated IL-6 levels and STAT3 activation are consistently observed in models of skeletal muscle atrophy [69]; and hyperactivation of the IL-6/STAT3 axis directly induces myofiber atrophy in mice [70].

The imbalance in local inflammatory homeostasis within muscle tissue, rather than the systemic cytokine milieu alone, underlies the link between inflammation and muscle injury [71]. Among the cytokines elevated in COPD, three have been mechanistically linked to skeletal muscle dysfunction. TNF-α activates NF-κB signaling to upregulate the E3 ubiquitin ligases MuRF1 and MAFbx, promoting proteasomal degradation of contractile proteins, while concurrently suppressing PGC-1α-mediated myogenesis [72,73]. IL-6, through STAT3, downregulates the myogenic regulators MyoD and myogenin, thereby inhibiting muscle differentiation [74,75]. Elevated IL-8 levels correlate with reduced muscle strength in COPD patients [76,77]. These findings collectively establish the major cytokine-driven pathways through which systemic inflammation compromises skeletal muscle function in COPD.

### 3.2. Oxidative Stress

Oxidative stress is a well-established feature of COPD pathophysiology [78]. In skeletal muscle, ROS originate from mitochondrial electron transport chain leakage, NADPH oxidase, and infiltrating inflammatory cells [79]. This elevated oxidative burden directly damages contractile proteins, impairs excitation–contraction coupling, and activates proteolytic systems. ROS and mitophagy engage in a bidirectional relationship: ROS trigger mitophagy as a compensatory response, whereas sustained oxidative pressure ultimately disables the mitophagic machinery, establishing a self-reinforcing loop that amplifies muscle damage (Figure 2).

#### 3.2.1. Source of ROS

Both systemic and local skeletal muscle oxidative stress are elevated in COPD patients [78]. ROS in skeletal muscle derive from three primary sources: mitochondrial respiration, sarcolemmal NADPH oxidase (NOX), and exogenous input from inflammatory cells.

Mitochondrial respiration is a major source of ROS production in skeletal muscle. Studies using mitochondria isolated from the quadriceps of COPD patients have detected H_2_O_2_ generation associated with complex I and complex II substrates, with complex III identified as the primary site due to the topology of its Q-cycle site [80,81].

Beyond the mitochondrion, NADPH oxidase on the sarcolemma constitutes a second major source. NOX in the endomysium is progressively activated under systemic inflammation and cigarette smoke exposure, primarily via the Rac1-p22 axis, assembling into a functional NOX2/4 complex that generates O_2_^−^• and H_2_O_2_ [82]. The resulting ROS engage the CaMKII-Ca^2+^-calpain cascade, weakening excitation–contraction coupling and activating proteases [83].

In addition to these intrinsic sources, infiltrating neutrophils and macrophages contribute exogenous ROS through respiratory burst. These cells release O_2_^−^, H_2_O_2_, and HOCl via membrane-bound NOX2 and myeloperoxidase, which subsequently diffuse to muscle fibers in a paracrine manner [52].

#### 3.2.2. ROS-Induced Skeletal Muscle Dysfunction

ROS from multiple sources induce widespread alterations in skeletal muscle. Protein-level changes, including protein carbonylation, lipid peroxidation, and nitration, are well documented in COPD muscle, whereas evidence for DNA damage remains limited [10,78]. The pathophysiological impact of oxidative stress can be understood at two levels: direct damage to contractile proteins, and signal amplification that engages multiple pathogenic pathways.

Direct damage: This involves oxidation of contractile proteins and excitation-contraction uncoupling. Oxidative stress is evident in COPD skeletal muscle even at a preclinical stage. ROS directly oxidize contractile proteins, including actin at the Cys374 residue and troponin, disrupting excitation–contraction coupling, attenuating myofiber responsiveness to neural impulses, and reducing endurance [84]. This phenomenon is particularly evident in diaphragm dysfunction induced by cigarette smoke exposure [79].

Signal amplification: This links multiple pathogenic pathways. As the disease progresses, oxidative stress gradually becomes a signal amplifier that undermines skeletal muscle function by engaging inflammation, proteolytic systems, protein synthesis inhibition, and impaired regeneration [78]. ROS activate NF-κB, promoting inflammatory cell infiltration and inducing skeletal muscle atrophy [85,86]. ROS-mediated activation of IL-6 and TNF-α represents a critical node in the feedback loop linking oxidative stress and inflammation [87]. Concurrently, ROS activate FoxO transcription factors, which upregulate the muscle-specific E3 ubiquitin ligases MuRF1 and MAFbx, driving proteasomal degradation [88,89,90]. High concentrations of H_2_O_2_ activate the LKB1/AMPK pathway and inhibit mTORC1, inducing autophagy, whereas elevated ROS levels also suppress the PI3K/AKT/mTOR signaling axis, reducing protein synthesis [91,92]. Furthermore, ROS deplete intracellular glutathione, leading to NF-κB activation and downregulation of MyoD and myogenin, thereby impairing myoblast differentiation and muscle regeneration [93,94,95]. Notably, NF-κB activation, FoxO upregulation, and autophagy induction intersect with mitophagy at multiple levels. These pathways can be both targets and regulators of mitochondrial quality control, a reciprocity that positions mitophagy as a key node through which oxidative stress amplifies its effects.

### 3.3. Hypoxia

Hypoxia is an inevitable consequence of impaired gas exchange in patients with long-term airway obstruction and is considered a contributing factor to skeletal muscle wasting in COPD. In animal models, chronic hypoxia directly induces muscle atrophy, closely mimicking the hypoxic microenvironment of skeletal muscle in COPD patients [96]. Multiple mechanisms underlie this effect. Hypoxia downregulates mTOR activity, leading to marked suppression of protein synthesis, while simultaneously activating the ubiquitin–proteasome system and autophagy [96] (Figure 2).

Transcriptional regulation by hypoxia-inducible factors plays a central role. HIF-1α mRNA levels are elevated in the quadriceps of COPD patients and are associated with activation of TNF-α signaling [97]. In vitro studies confirm that HIF-1α expression increases in hypoxic C2C12 myotubes, and canonical Wnt signaling involved in muscle regeneration is suppressed under hypoxia [98]. HIF-2α also contributes to chronic hypoxia-induced skeletal muscle atrophy, as specific knockout of HIF-2α abolishes the detrimental effects of long-term hypoxia on muscle proliferation, an effect mediated by angiotensin-converting enzyme signaling [99]. In addition, hypoxia interferes with myogenesis by reducing myogenin and MyoD1 expression, impairing the terminal maturation of myotubes and suppressing muscle satellite cell proliferation [100,101,102]. Collectively, these observations establish hypoxia as a direct driver of skeletal muscle dysfunction through suppression of protein synthesis, activation of proteolytic pathways, and impairment of muscle regeneration. Hypoxia also engages the mitophagy machinery through HIF-1α-dependent upregulation of BNIP3/NIX and FUNDC1, linking the hypoxic stress response to mitochondrial quality control.

### 3.4. Aging

Aging is a significant contributor to skeletal muscle dysfunction in COPD, a notion supported by comparisons between healthy individuals and COPD patients across different age groups (Figure 2). Compared with young individuals, the elderly exhibit markedly reduced muscle mass and strength, accompanied by alterations in fiber-type composition and increased fat and connective tissue content.

During aging-induced muscle atrophy, the balance between protein synthesis and degradation is disrupted, with degradation predominating. Three major proteolytic pathways are activated in aged skeletal muscle: the ubiquitin–proteasome system (UPS), the autophagy–lysosome pathway (ALP), and apoptosis [103]. In the UPS, muscle-specific E3 ubiquitin ligases MuRF1 and Atrogin-1 are upregulated in aging models, promoting proteasomal degradation of contractile proteins [104]. In the ALP, upstream regulators FOXO3 and TFEB are activated, supporting increased expression of autophagy-related genes [105]. However, protein marker analysis reveals that aged skeletal muscle primarily exhibits autophagic flux disturbance, characterized by accumulation of Beclin-1 and LC3-II without corresponding increases in the transcript levels of upstream autophagy genes [106]. Notably, this autophagic disturbance is fiber-type-specific: autophagic markers are maintained in slow-twitch fibers but lost in fast-twitch fibers [107]. Given that mitochondria are major targets of ALP clearance, this autophagic flux disturbance suggests impaired mitophagy in aged skeletal muscle. In addition, aging skeletal muscle exhibits increased apoptosis, with activation of calpain and caspase cascades that act synergistically with the UPS.

The aging process in COPD skeletal muscle is further accelerated by adverse stimuli such as inflammation and oxidative stress, creating a complex pathological microenvironment in which multiple regulatory factors interact. Recent studies have revealed that BNIP3, a mitophagy receptor, is upregulated in aged skeletal muscle and coordinates both mitochondrial and lysosomal homeostasis [108]. These findings provide a foundation for understanding how aging intersects with mitochondrial quality control in COPD muscle dysfunction.

## 4. Mitophagy as a Central Hub in the Multifactorial Pathogenesis of COPD Skeletal Muscle Dysfunction

The preceding sections established that inflammation, oxidative stress, hypoxia, and aging each contribute to skeletal muscle dysfunction through distinct mechanisms. Each of the four drivers impinges on mitochondria, albeit through distinct proximal signals. Whether they converge on a common quality-control pathway is the question that follows, and the evidence reviewed below suggests that they do.

### 4.1. Mitophagy & Systemic Inflammation

Systemic chronic inflammation is a core driver of skeletal muscle dysfunction in COPD, and emerging evidence positions mitophagy as an active regulator of this inflammatory network rather than a passive downstream event. The interplay between mitophagy and inflammation operates at two interconnected levels: within immune cells, where mitophagy determines the quality and intensity of the inflammatory output, and within myofibers, where inflammatory signals directly impinge on mitochondrial quality control. These two levels coalesce into a bidirectional feedback loop that sustains and amplifies inflammation.

Mitophagy regulates immune cell function. In macrophages, mitophagy status directly dictates polarization direction. Macrophages with intact PINK1/Parkin-mediated mitophagy tend toward an M2 anti-inflammatory phenotype, clearing damaged mitochondria and limiting NLRP3 inflammasome activation [109,110]. Conversely, defective mitophagy locks macrophages in M1 pro-inflammatory polarization, triggering mitochondrial ROS bursts and excessive release of IL-6, TNF-α, and IL-1β [111]. PINK1 expression is reduced in COPD patient alveolar lavage fluid and correlates with an elevated M1/M2 ratio [112]. Histone lactylation has also been shown to regulate PRKN-mediated mitophagy and promote M2 polarization [113]. In neutrophils, mitophagy is critically involved in the formation of neutrophil extracellular traps. A subset of neutrophils relies on SIRT1-mediated mitophagy to release mitochondrial DNA and form active NETs [114]. Once formed, NETs can in turn impair FUNDC1-dependent mitophagy through phosphorylation of FUNDC1 at Tyr18, triggering mitochondrial quality control imbalance, ROS overproduction, and ferroptosis [115]. Inhibition of TLR4 or RIPK3 relieves this restriction on mitophagy and reduces NET-driven necroptosis [116]. In T cells and NK cells, appropriate levels of mitophagy are essential for maintaining mitochondrial fitness and supporting cell differentiation and survival. Activating BNIP3-mediated mitophagy enhances CD8^+^ T cell survival [117], and mitophagy is required for the transition of thymocytes to peripheral naïve T cells and for the maturation of invariant NK cells [118,119]. Suppression of mitophagy leads to accumulation of damaged mitochondria and apoptosis in proliferating NK cells. Collectively, defective mitophagy in immune cells represents an intrinsic cellular mechanism that amplifies inflammation: immune cells with impaired mitochondrial quality control themselves become drivers of the inflammatory milieu.

Inflammatory cytokines directly act on myofibers to suppress mitophagy. Beyond their effects on immune cells, circulating pro-inflammatory cytokines directly modulate mitophagy in skeletal muscle. TNF-α, IL-6, and IL-1β suppress the expression of PINK1 and Parkin through NF-κB and STAT3 signaling, thereby attenuating the ubiquitin-dependent mitophagy pathway [120,121]. In COPD patients, quadriceps muscle exhibits elevated protein levels of BNIP3L and FUNDC1, with BNIP3L expression correlating positively with plasma CRP levels [122]. Notably, this same study reported elevated Parkin protein alongside reduced PINK1, with mRNA levels unchanged [122], suggesting a compensatory but inefficient activation state. Whether this reflects enhanced mitophagic flux or impaired degradation cannot be resolved by static markers alone. In animal models, LPS injection induces dynamic changes in mitophagy markers in skeletal muscle, with initial activation followed by relative suppression over time [123]. These findings establish a direct mechanistic link between systemic inflammation and impaired mitochondrial quality control in muscle fibers.

**Figure 3:**
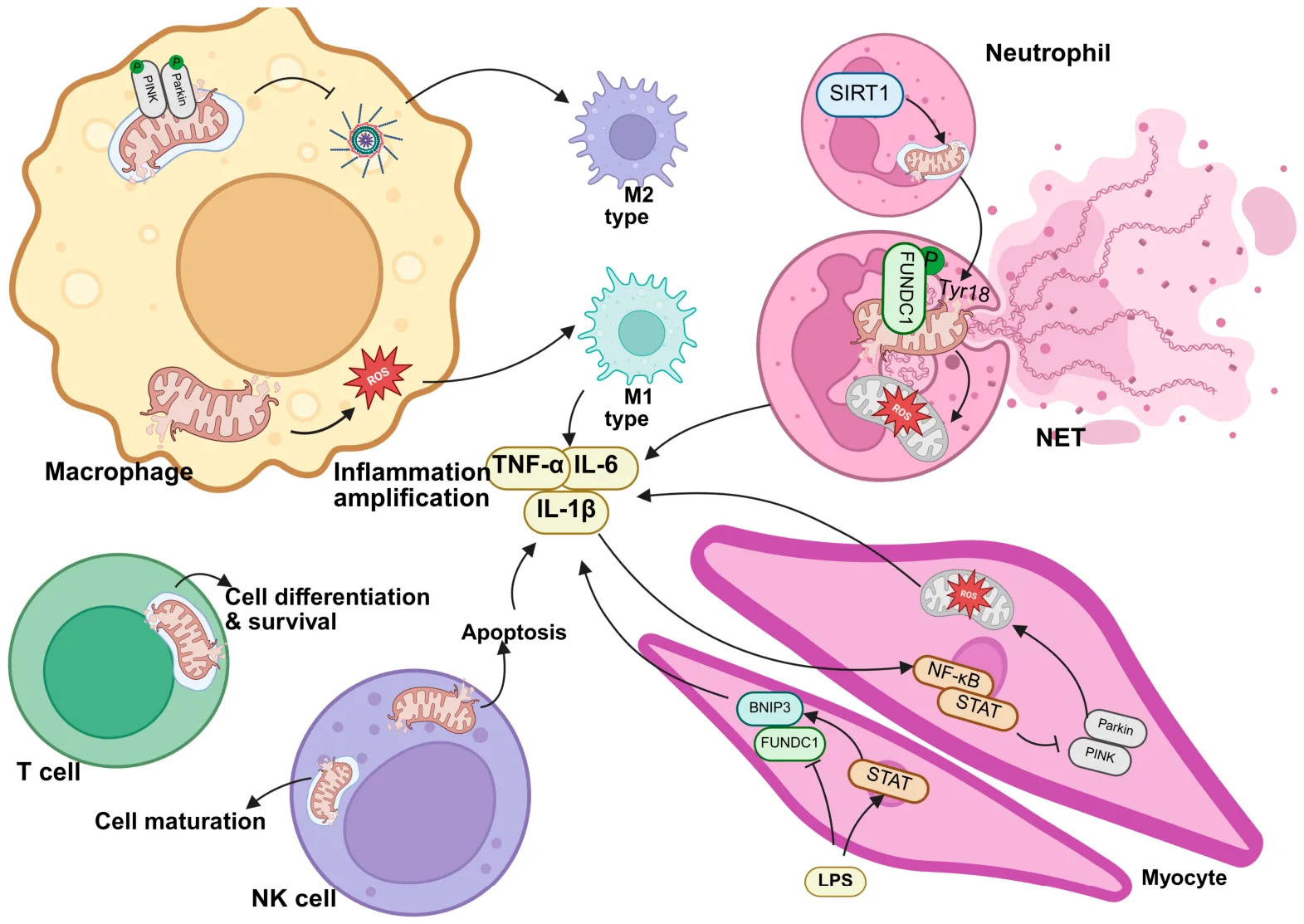
Mitophagy and systemic inflammation. Mitophagy regulates three types of immune cells, indirectly affecting skeletal muscle. It controls macrophage polarization via the NF-κB-NLRP3 axis, modulates neutrophil extracellular trap (NET) formation, and supports T cell and natural killer (NK) cell survival. In addition, local inflammatory signals directly act on myofiber mitophagy, establishing a bidirectional regulatory network. This figure panel was created in BioRender. Li, J. (2026) https://BioRender.com/l489zgr (accessed on 28 September 2026). Abbreviations: NF-κB, nuclear factor kappa-light-chain-enhancer of activated B cells; NLRP3, NOD-like receptor family pyrin domain-containing 3; NET, neutrophil extracellular trap; Tyr18, tyrosine residue 18 of FUNDC1; NK, natural killer; LPS, lipopolysaccharide; SIRT1, Sirtuin 1.

Together, these two levels of crosstalk form a self-reinforcing circuit. Inflammatory cytokines suppress mitophagy in myofibers, leading to the accumulation of damaged mitochondria and the release of mitochondrial ROS and pro-inflammatory signals, which, in turn, sustain immune cell activation and further cytokine production. Immune cells with defective mitophagy amplify the inflammatory output, perpetuating the cycle. This loop tightens with each iteration: defective myofiber mitophagy fuels immune activation, whose cytokine output further compromises the clearance machinery (Figure 3).

### 4.2. Oxidative Stress

**Figure 4:**
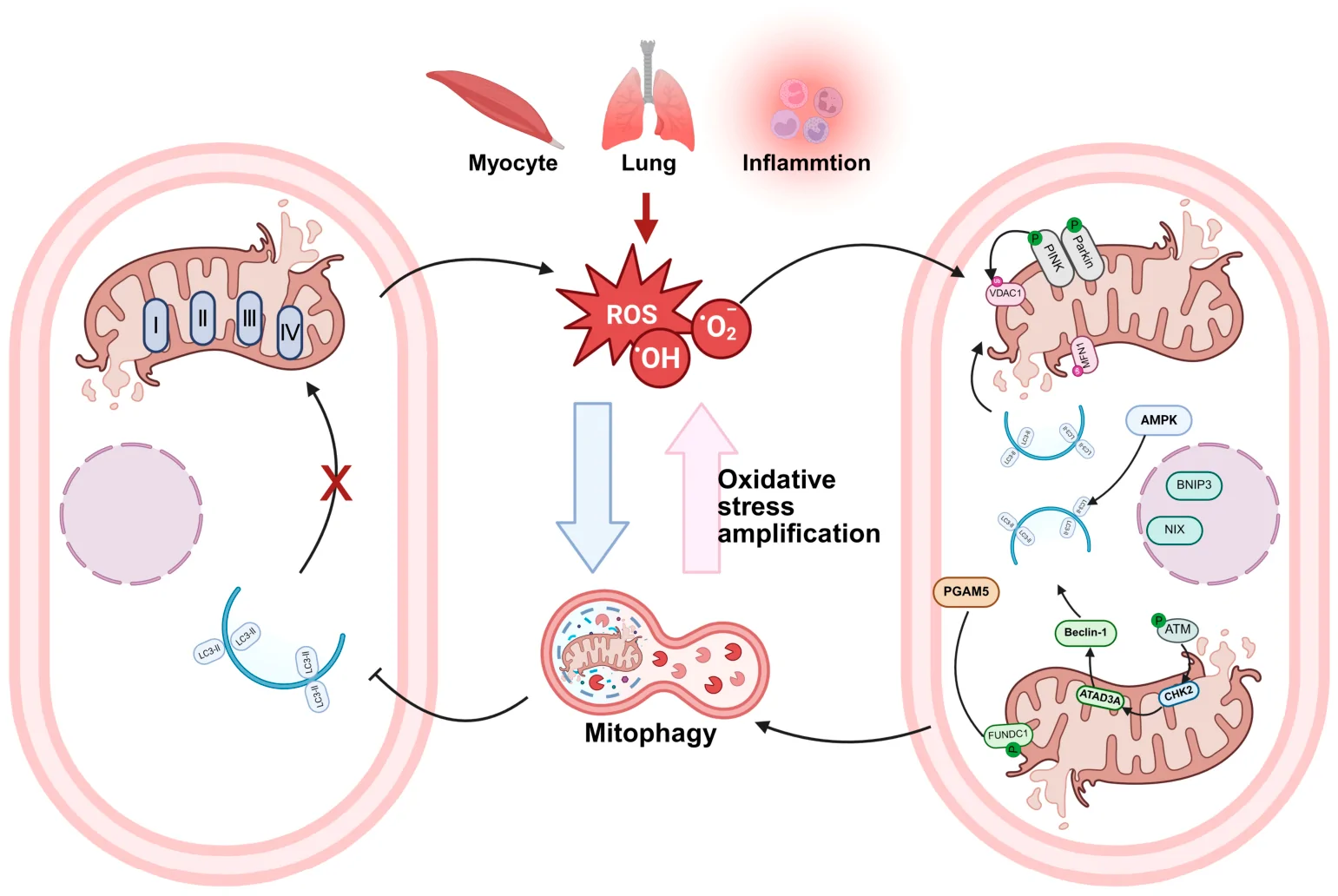
Mitophagy and oxidative stress. Mitophagy and oxidative stress form a bidirectional regulatory network. Defective mitophagy leads to accumulation of damaged mitochondria, which continuously generate ROS and cause oxidative damage to skeletal muscle. Conversely, oxidative stress activates mitophagy through multiple pathways, including the PINK1/Parkin axis, the ATM-CHEK2 axis, the AMPK pathway, and the BNIP3/NIX axis. This figure panel was created in BioRender. Li, J. (2026) https://BioRender.com/l489zgr (accessed on 28 September 2026). Abbreviations: ROS, reactive oxygen species; PINK1, PTEN-induced putative kinase 1; Parkin, E3 ubiquitin-protein ligase (PRKN); ATM, ataxia-telangiectasia mutated; CHEK2, checkpoint kinase 2; AMPK, AMP-activated protein kinase; BNIP3, BCL2-interacting protein 3; NIX, BNIP3-like protein (BNIP3L); COPD, chronic obstructive pulmonary disease.

Oxidative stress and mitophagy are engaged in a bidirectional relationship that forms a cyclical regulatory network in COPD skeletal muscle (Figure 4). Defective mitophagy leads to the accumulation of damaged mitochondria, which become a major source of ROS. Conversely, ROS trigger mitophagy as a compensatory response, yet sustained oxidative pressure ultimately overwhelms this system. Together, these two directions create a self-reinforcing loop that amplifies mitochondrial dysfunction and muscle damage.

Defective mitophagy is a major source of oxidative stress. In healthy skeletal muscle, clearance of damaged mitochondria depends on PINK1/Parkin-mediated mitophagy [124]. When this machinery fails, damaged mitochondria accumulate. Due to structural and functional abnormalities in electron transport chain complexes I and III, these dysfunctional mitochondria exhibit substantial electron leakage, generating O_2_^−^• and H_2_O_2_ [125]. Clinical evidence supports this mechanism: mitochondrial density is reduced in the quadriceps of COPD patients, yet oxidative stress markers are markedly elevated, indicating that remaining damaged mitochondria have a higher ROS-producing capacity [80,126]. Beyond this local source, infiltrating immune cells contribute an exogenous supply of ROS. Alveolar macrophages in COPD patients are markedly increased in number and release both inflammatory cytokines and ROS upon activation, while neutrophils in the peripheral blood and lung tissue have also been confirmed as sources of ROS [127,128]. These systemic ROS sources synergize with mitochondria-derived ROS, collectively amplifying the oxidative burden on skeletal muscle.

Oxidative stress initiates and, when sustained, exhausts mitophagy. Mitochondrial ROS triggers mitophagy through multiple pathways. The classical PINK1/Parkin pathway is activated when ROS cause a drop in mitochondrial membrane potential, stabilizing PINK1 on the outer membrane and recruiting Parkin to ubiquitinate mitochondrial surface proteins [129]. Recent studies have identified an additional ROS-sensing axis involving ATM-CHK2 (ataxia-telangiectasia mutated–checkpoint kinase 2), which promotes mitophagy through coordinated phosphorylation of ATAD3A, OPTN, and Beclin-1 [130]. ROS also activate AMPK signaling, which relieves mTORC1-mediated inhibition of ULK1 and initiates autophagic signaling [131]. Receptor-mediated mitophagy is similarly regulated by oxidative stress: ROS activate PGAM5 to dephosphorylate FUNDC1, enhancing its binding to LC3 [33], and stabilize HIF-1α to upregulate BNIP3/NIX transcription, mediating ubiquitin-independent mitophagy [132]. Notably, ROS itself promotes Parkin recruitment through VDAC1, which regulates mtROS flux into the cytosol [133]. While these ROS-triggered mitophagic responses serve a compensatory role in the short term, sustained oxidative stress ultimately leads to oxidative modification and inactivation of key mitophagy components, shifting the balance from compensation to failure.

Together, these two directions form a self-perpetuating cycle. Defective mitophagy allows damaged mitochondria to accumulate and release excessive ROS; these ROS in turn trigger further mitophagy as a compensatory attempt, but chronic exposure exhausts the system and accelerates mitochondrial deterioration. This vicious cycle, in which mitophagy dysfunction and oxidative stress mutually reinforce each other, lies at the heart of the amplificatory network that drives COPD skeletal muscle dysfunction (Figure 4).

### 4.3. Hypoxia

Direct evidence linking hypoxia to mitophagy in COPD skeletal muscle remains limited, yet accumulating data from cellular and animal models point to a bidirectional crosstalk between the two. Hypoxia triggers mitophagy as an acute adaptive response, whereas dysfunctional mitophagy, in turn, exacerbates tissue hypoxia. These two directions form a potential feedback loop that, under chronic conditions, may shift from compensation to pathology.

Hypoxia triggers mitophagy through multiple pathways. The HIF-1α-BNIP3/NIX axis is the best-characterized mechanism. Under hypoxic conditions, stabilized HIF-1α translocates to the nucleus and binds to the hypoxia response element in the BNIP3 promoter, initiating its transcription [30]. A recent quantitative intravital imaging study identified BNIP3 as the master regulator of hypoxia-induced mitophagy in vertebrate muscle, with deletion of NIX, FUNDC1, PINK1, or Parkin having only marginal effects [134]. Hypoxia also rapidly activates mitophagy through the FUNDC1 pathway via a phosphorylation switch: dephosphorylation of FUNDC1 at Ser13 by PGAM5, coupled with phosphorylation at Ser17 by ULK1, relieves inhibition of FUNDC1-LC3 interaction and allows efficient autophagosome targeting [32]. The role of the PINK1/Parkin pathway in hypoxia-induced skeletal muscle mitophagy remains less clear. Evidence from non-skeletal muscle tissues has yielded inconsistent results, and direct evidence in skeletal muscle is still lacking. However, given that oxidative stress is elevated under hypoxia and that PINK1/Parkin is a well-established ROS-sensitive pathway, its involvement in hypoxic mitophagy cannot be excluded and warrants further investigation using skeletal muscle-specific models.

Dysfunctional mitophagy, in turn, exacerbates the hypoxic microenvironment. Beyond the unidirectional activation of mitophagy by hypoxia, the reverse relationship is equally important. Excessive or dysfunctional mitophagy can itself induce or worsen local hypoxia through multiple mechanisms, including mitochondrial electron leakage with excessive ROS production, metabolic reprogramming with impaired oxidative phosphorylation, and the amplification of inflammatory cycles [135]. These factors collectively create a self-reinforcing cycle: hypoxia triggers mitophagy as a compensatory attempt, but when the system is overwhelmed, dysfunctional mitophagy leads to mitochondrial damage and further exacerbation of hypoxia.

**Figure 5:**
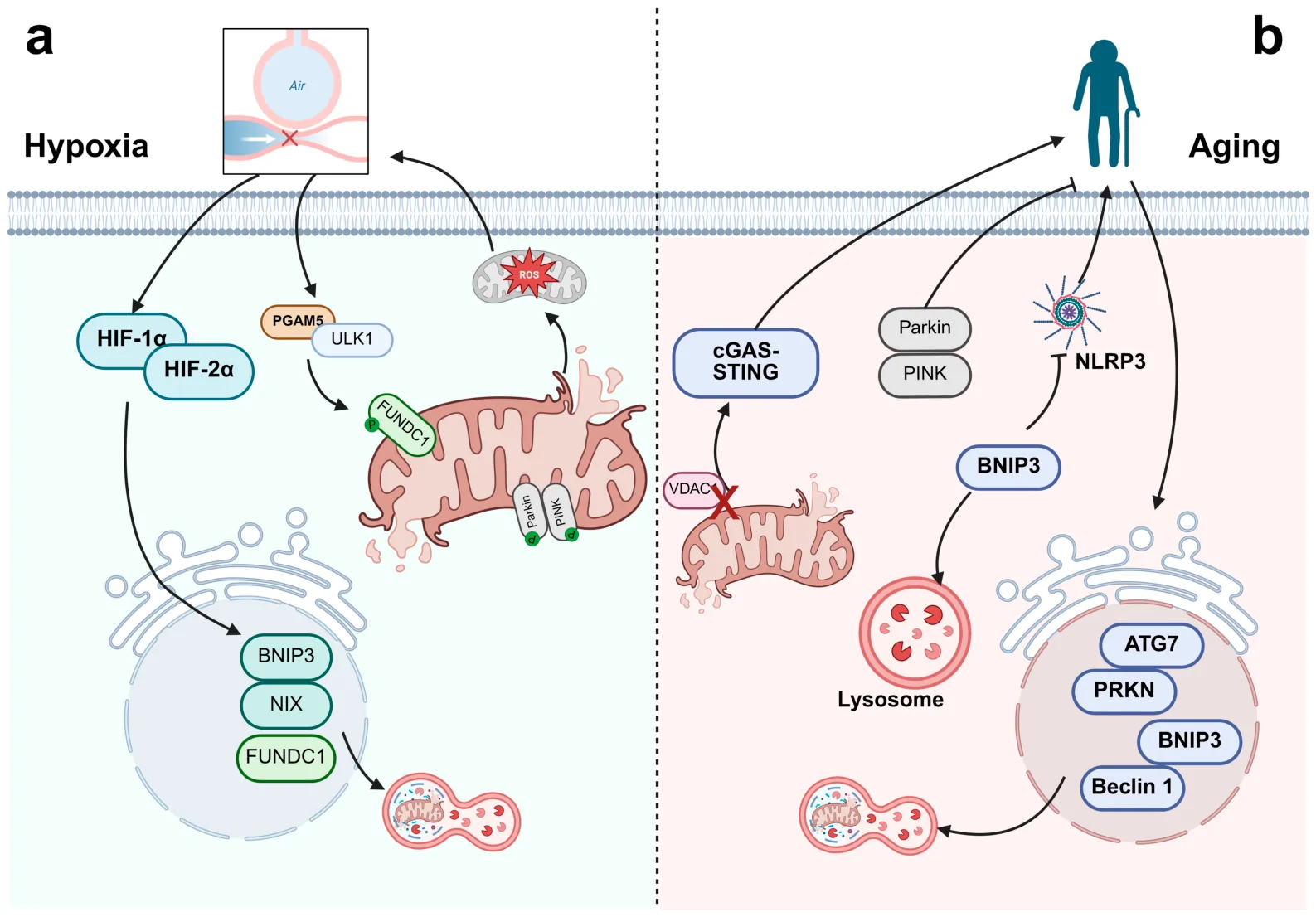
Mitophagy in hypoxia and aging. In hypoxia (**a**), the HIF-1α-BNIP3/NIX axis and FUNDC1 phosphorylation switch trigger mitophagy as an acute adaptive response, but chronic hypoxia compromises lysosomal acidification and autophagic flux, shifting the balance from clearance to failure; dysfunctional mitophagy in turn exacerbates tissue hypoxia through ROS production and metabolic disruption. In aging (**b**), mitophagy declines through reduced expression of PRKN, ATG7, BECN1, and BNIP3, with BNIP3 showing compensatory upregulation in some contexts, suggesting a dynamic transition from compensation to decompensated decline. Lysosomal dysfunction is a critical bottleneck, as acidification and proteolytic capacity decline with age, stalling autophagic flux; BNIP3 deficiency impairs both mitochondrial tagging and lysosomal homeostasis, triggering NLRP3-mediated inflammation. Defective mitophagy promotes VDAC1 oligomerization, mtDNA release, and cGAS-STING activation, accelerating the senescence-associated secretory phenotype. This figure panel was created in BioRender. Li, J. (2026) https://BioRender.com/l489zgr (accessed on 28 September 2026). Abbreviations: HIF, hypoxia-inducible factor; BNIP, BCL2-interacting protein; NIX, BNIP3-like protein; FUNDC1, FUN14 domain-containing protein 1; ROS, reactive oxygen species; PRKN, Parkin gene; ATG7, autophagy-related gene 7; BECN1, Beclin 1 gene; VDAC1, voltage-dependent anion channel 1; cGAS-STING, cyclic GMP-AMP synthase/stimulator of interferon genes; NLRP3, NOD-like receptor family pyrin domain-containing 3; The ‘X’ symbol indicates a blocked or impaired step in the pathway.

Thus, hypoxia and mitophagy form a bidirectional regulatory loop in COPD skeletal muscle (Figure 5a). Hypoxia activates mitophagy through the HIF-1α-BNIP3/NIX axis and the FUNDC1 phosphorylation switch, with additional involvement of the PINK1/Parkin pathway. Conversely, dysfunctional mitophagy aggravates tissue hypoxia through ROS production and metabolic disruption. Under acute conditions, this loop may serve a protective role by clearing damaged mitochondria. However, in the setting of chronic COPD, persistent hypoxic stress and accumulating mitochondrial damage overwhelm the clearance capacity, shifting the loop from a compensatory mechanism to a pathological amplifier that sustains and worsens mitochondrial dysfunction.

### 4.4. Aging

In human skeletal muscle, mitochondrial dysfunction is a hallmark of aging and significantly impairs muscle function [136]. A core feature of age-related mitochondrial dysfunction is insufficient mitophagy, which, in turn, hinders mitochondrial biogenesis [137]. Multiple lines of evidence indicate that muscle aging leads to reduced mitophagy. In aged women, the expression of PRKN, ATG7, BECN1, and BNIP3 is decreased in skeletal muscle [138]; muscle biopsies from elderly men confirm this trend [37]. However, some reports have found increased mitophagy in aged skeletal muscle, with BNIP3 showing compensatory upregulation and coordinating mitochondrial and lysosomal functions to maintain mitophagy homeostasis [108]. This apparent contradiction suggests that mitophagy may undergo a dynamic transition from compensatory enhancement to decompensated decline during aging, since compensatory BNIP3 upregulation may be insufficient to overcome the lysosomal degradative bottleneck.

Conversely, defective mitophagy accelerates the aging process. In prematurely aged myofibers, impaired mitophagy leads to increased release of mitochondrial DNA through VDAC1 oligomerization, activating the cGAS-STING pathway and the senescence-associated secretory phenotype [139]. Treatment with VBIT4, an inhibitor of VDAC1 oligomerization, reduces mtDNA release and suppresses cGAS-STING activation, confirming the mechanistic link between mitophagy failure and mtDNA-driven inflammation. Functional evidence further supports this bidirectional relationship. Knockout of PINK1 or Parkin induces a premature aging-like phenotype in adult skeletal muscle [140], whereas Parkin overexpression increases mitochondrial enzyme activity and mitochondrial content in aged mouse muscle and alleviates age-associated oxidative stress [141]. Loss of BNIP3 leads to mitochondrial dysfunction, reduced autophagic flux, and lysosomal impairment, which, in turn, activates TLR9-dependent inflammation and the NLRP3 inflammasome [108]. These observations collectively indicate that defective mitophagy not only accompanies but actively drives the aging process in skeletal muscle, primarily through mtDNA-mediated inflammatory signaling and the disruption of mitochondrial–lysosomal crosstalk.

Beyond reduced receptor expression, lysosomal dysfunction is a critical bottleneck in aged muscle [142]. Lysosomal acidification and proteolytic capacity decline with age, stalling autophagic flux even when mitochondria are successfully sequestered [143]. BNIP3 embodies this duality: it tags mitochondria for clearance while also coordinating lysosomal homeostasis; its deficiency impairs both processes and triggers TLR9/NLRP3-mediated inflammation [144]. Thus, age-related mitophagy failure reflects convergent deterioration of cargo tagging and lysosomal disposal, a dual deficit that lowers the threshold for quality-control failure and feeds the self-amplifying vortex (Figure 5b).

At the molecular level, lysosomal degradation capacity in aged muscle is compromised by a feed-forward cascade initiated by impaired TFEB signaling. TFEB, the master transcriptional regulator of lysosomal biogenesis, drives the expression of v-ATPase subunits and other lysosomal genes [145]. In aged skeletal muscle, TFEB nuclear translocation is diminished, reducing v-ATPase subunit transcription and limiting the assembly of functional proton pumps [146]. Notably, total TFEB protein levels may be elevated in aged muscle while nuclear translocation is diminished, suggesting that increased lysosomal protein content may reflect impaired clearance rather than enhanced biogenesis. Compounding this transcriptional deficit, mitochondrial protein import stress causes disassembly of existing v-ATPase complexes, leading to vacuolar deacidification and impaired hydrolytic enzyme activity [147]. As degradation stalls, lipofuscin progressively accumulates within lysosomes, and its components can further inhibit v-ATPase activity, establishing a self-reinforcing cycle of lysosomal failure [148,149]. Together, these lesions constitute the molecular basis of the lysosomal bottleneck that sustains the mitophagy vortex.

### 4.5. Integration: Mitophagy as a Network Amplifier in COPD Skeletal Muscle

Pathophysiologically, this network converges into a self-sustaining disequilibrium centered on the ratio between mitochondrial injury load and lysosomal disposal capacity. The numerator, driven by inflammation and oxidative stress, persistently escalates, whereas the denominator, constrained by hypoxia and aging, undergoes progressive attenuation. Once this ratio exceeds a critical threshold, retained damaged mitochondria liberate mitochondrial DNA and overproduce superoxide, both of which amplify upstream inflammatory signals and further impair lysosomal acidification, thereby eroding the very machinery required for their own elimination. This self-amplifying loop, in which a failure in quality control exacerbates its own precipitating factors, rather than a spatially predefined compartmentalization, constitutes the operational basis of what we term the mitophagy vortex in COPD skeletal muscle pathology.

This vortex is not sustained by a single linear pathway but is reinforced by two interconnected patterns of amplification emerging from the evidence. The first is the inflammatory–oxidative amplification axis: inflammation suppresses mitophagy, leading to accumulation of damaged mitochondria and ROS burst; these ROS, in turn, activate NF-κB and amplify inflammation, which further suppresses mitophagy. In this axis, mitophagy failure sits at the center of a self-perpetuating loop between inflammation and oxidative stress. The second is the hypoxic–aging clearance impairment axis: hypoxia and aging compromise the execution phase of mitophagy by impairing lysosomal function and autophagic flux, thereby lowering the threshold for mitochondrial quality control failure. This axis does not drive the production of damaged mitochondria; rather, it impairs their disposal, making the system more vulnerable to the inflammatory–oxidative burden generated by the first axis.

These two axes reinforce each other. The inflammatory–oxidative axis generates ROS that further damage lysosomal function, exacerbating the clearance impairment. The clearance impairment, in turn, allows damaged mitochondria to persist, producing more ROS and fueling the inflammatory–oxidative axis. The net effect is a self-accelerating process, one that can be described as a mitophagy vortex, in which mitochondrial quality control progressively deteriorates, and the four pathogenic factors become increasingly intertwined. This integrated view explains why single-target interventions have had limited success, and why restoring mitochondrial quality control at the level of mitophagy may offer a more effective strategy for breaking the pathological network.

### 4.6. Therapeutic Targeting of Mitophagy: From Concept to Intervention

The integrated view presented above identifies several points at which intervention might be possible. Currently, however, no pharmacotherapy is approved for skeletal muscle dysfunction in COPD, a gap noted at the outset of this review. Whether restoring mitophagy could address this gap is not yet known, but the available evidence offers some direction.

Direct evidence from COPD models: Ito et al. [150] demonstrated that insufficient Parkin-mediated mitophagy drives skeletal muscle atrophy in COPD through mitochondrial ROS accumulation and MuRF-1-mediated myosin heavy chain degradation. This effect was consistently observed across cigarette smoke-exposed myotubes, Parkin^^−^/^−^^ mice, and muscle biopsies from COPD patients with sarcopenia. These findings establish PINK1/Parkin as a functionally relevant pathway in COPD skeletal muscle.

Evidence from aging and related models: Studies in aging models have shown that enhancing PINK1/Parkin activity can preserve mitochondrial quality control, while BNIP3 deficiency exacerbates muscle inflammation and atrophy through TLR9/NLRP3 activation [108,140,141]. These findings provide indirect support for mitophagy-targeted strategies in conditions where aging is a contributing factor, though direct evidence in COPD muscle is lacking.

Blocking downstream inflammatory consequences. Defective mitophagy triggers inflammatory pathways through VDAC1 oligomerization and NLRP3 activation [109,110,139]. VBIT4, a VDAC1 oligomerization inhibitor, reduces mtDNA release and cGAS-STING activation in aged myofibers [139]. NLRP3 inhibitors have shown efficacy in cancer cachexia and diabetic models but remain untested in COPD muscle, and MCC950 was discontinued due to hepatotoxicity [151,152]. These downstream inhibitors may complement, but cannot replace, strategies that restore mitophagy itself.

Urolithin A activates mitophagy through PINK1-Parkin-dependent and independent pathways. In a 4-month randomized trial in middle-aged adults, supplementation improved muscle strength and aerobic endurance, with parallel increases in mitophagy-related proteins in skeletal muscle [153]. A separate randomized trial in adults aged 65–90 years found that UA improved muscle endurance at 2 months but did not significantly improve maximal ATP production at 4 months or 6-minute walk distance, indicating that molecular activation of mitophagy does not automatically translate into functional improvement [154]. A 2026 systematic review of five RCTs found that the evidence base remains limited to small, short-term trials; effects on the 6-minute walk test were directionally favorable but statistically inconclusive [155]. This decoupling between molecular activation and functional outcome suggests that enhancing mitophagy initiation alone may be insufficient to improve muscle function in aging or disease states, where impaired lysosomal clearance capacity may limit the conversion of autophagic signals into functional benefit. Even so, these human data establish proof of principle that pharmacological activation of mitophagy is achievable in humans, a prerequisite for any eventual translation to COPD.

However, several practical challenges temper the translational promise of mitophagy-enhancing agents in COPD. Patients with advanced COPD are typically elderly, frail, and receive multiple medications, raising concerns about drug interactions and tolerability. Yet, whether mitophagy-enhancing agents retain efficacy and safety in this complex inflammatory and metabolic milieu remains largely unstudied. Although UA reaches skeletal muscle and activates mitophagy at the molecular level [153], functional improvements remain inconsistent [154], which may partly reflect insufficient target-site exposure. To address this, muscle-specific delivery systems, such as MyomP1-modified lipid nanoparticles, could enhance local drug concentration and improve the therapeutic index [156], a direction that has shown early preclinical feasibility and warrants further investigation in the context of COPD muscle dysfunction. More fundamentally, it remains unclear whether pharmacological enhancement of mitophagy can reverse an established vortex or whether intervention must occur earlier when clearance capacity is only partially compromised. Given that lysosomal dysfunction is a rate-limiting step in the vortex, agents that simultaneously enhance autophagic flux and restore lysosomal acidification, targeting both the numerator and denominator of the injury-to-clearance ratio, may be required to achieve a therapeutic threshold in advanced disease. Collectively, these challenges reflect a broader translational gap: most mitophagy-targeting strategies remain in preclinical stages, and their efficacy and safety in COPD patients with established muscle dysfunction have yet to be established.

The evidence for mitophagy-targeted interventions in COPD skeletal muscle is largely indirect. Most comes from aging models, muscular dystrophy, or toxin-induced damage; direct data from COPD models are sparse, with the exception of Ito et al.’s Parkin-deficient mouse work. Beyond the lack of COPD-specific evidence, the approach itself poses difficulties. Systemic modulation of mitophagy could affect heart, brain, or immune cells unpredictably, making muscle specificity a major challenge. The timing of intervention is equally uncertain. What works during early compensatory stages may not apply once failure is established. Mitophagy is not uniformly protective; its role depends on context and duration. These barriers are substantial, but they do not undermine the rationale. The network-based view outlined in this review still suggests that restoring mitophagic flux, rather than targeting individual cytokines or ROS scavengers, offers a more coherent strategy for breaking the pathological cycle.

## 5. Conclusions

COPD skeletal muscle dysfunction arises from the convergence of four pathogenic drivers, inflammation, oxidative stress, hypoxia, and aging, each of which impinges on mitophagy at a distinct step. Their convergence is not additive but multiplicative, operating through a single quality-control node where injury load, driven by inflammation and oxidative stress, progressively outruns clearance capacity, constrained by hypoxia and aging. Once this threshold is crossed, retained damaged mitochondria release mtDNA and ROS that amplify upstream inflammatory signals and further impair lysosomal function, establishing a positive feedback loop that tightens with each iteration. This is the mitophagy vortex, a self-accelerating state in which failing clearance and mounting damage progressively drive the system toward functional decline, and in which interventions targeting single upstream factors are inherently insufficient to reset the pathological network. Restoring the balance between mitochondrial injury and lysosomal clearance, whether by enhancing autophagic flux or preserving lysosomal function, may offer a more coherent therapeutic strategy.

## Data Availability

No new data were created or analyzed in this study. Data sharing is not applicable to this article.

## Abbreviations

The following abbreviations are used in this manuscript:

COPD	Chronic obstructive pulmonary disease
ATP	Adenosine triphosphate
ROS	Reactive oxygen species
NOX	NADPH oxidase
LC3-II	Lipidated form of microtubule-associated protein 1 light chain 3
UPS	Ubiquitin–proteasome system
ALP	Autophagy–lysosome pathway
FoxO	Forkhead box O (transcription factor)
MuRF1	Muscle RING finger 1
MAFbx	Muscle atrophy F-box (also Atrogin-1)
GSH	Glutathione
MyoD	Myogenic differentiation
HIF	Hypoxia-inducible factor
PINK1	PTEN-induced putative kinase 1
Parkin	E3 ubiquitin ligase (PRKN)
BNIP3	BCL2-interacting protein 3
NIX	BNIP3-like protein (BNIP3L)
FUNDC1	FUN14 domain-containing protein 1
AMPK	AMP-activated protein kinase
mTORC1	Mechanistic target of rapamycin complex 1
ULK1	Unc 51-like kinase 1
ATG	Autophagy-related gene
PI3P	Phosphatidylinositol 3 phosphate
NF-κB	Nuclear factor kappa-light-chain-enhancer of activated B cells
STAT3	Signal transducer and activator of transcription 3
JAK	Janus kinase
IL	Interleukin
TNF	Tumor necrosis factor
CRP	C-reactive protein
TLR4	Toll-like receptor 4
NET	Neutrophil extracellular trap
SIRT1	Sirtuin 1
TBK1	TANK-binding kinase 1
PGAM5	Phosphoglycerate mutase family member 5
ATM	Ataxia-telangiectasia mutated
CHEK2	Checkpoint kinase 2
cGAS	Cyclic GMP-AMP synthase
STING	Stimulator of interferon genes
